# Supplementary material for: The fluidic memristor as a collective phenomenon in elastohydrodynamic networks
Source: Nat Commun. 2024 Apr 10;15:3121. doi: 10.1038/s41467-024-47110-0 (PMC11006656; doi:10.1038/s41467-024-47110-0)
Supplement: Supplementary file 1 — Supplementary Information [file 41467_2024_47110_MOESM1_ESM.pdf]

Supplementary Information: 'The fluidic memristor as a collective phenomenon in elastohydrodynamic networks'

Alejandro Martínez-Calvo[1,2†], Matthew D. Biviano[3†], Anneline H. Christensen[3], Eleni Katifori[4,5], Kaare H. Jensen[3] and Miguel Ruiz-García[6,7,8*]

[1]Princeton Center for Theoretical Science, Princeton University, Princeton, NJ 08544, USA.
[2]Department of Chemical and Biological Engineering, Princeton University, Princeton, NJ 08544, USA.
[3]Department of Physics, Technical University of Denmark, DK 2800, Kgs. Lyngby, Denmark.
[4]Department of Physics and Astronomy, University of Pennsylvania, Philadelphia, Pennsylvania 19104, USA.
[5]Center for Computational Biology, Flatiron Institute, New York, NY 10010, USA.
[6]Departamento de Estructura de la Materia, Física Térmica y Electrónica, Universidad Complutense Madrid, 28040 Madrid, Spain.
[7]GISC - Grupo Interdisciplinar de Sistemas Complejos, Universidad Complutense Madrid, 28040 Madrid, Spain.
[8]Department of Mathematics, Universidad Carlos III de Madrid, 28911 Leganés, Spain.

*Corresponding author(s). E-mail(s): miguel.ruiz.garcia@ucm.es;
†These authors contributed equally to this work.

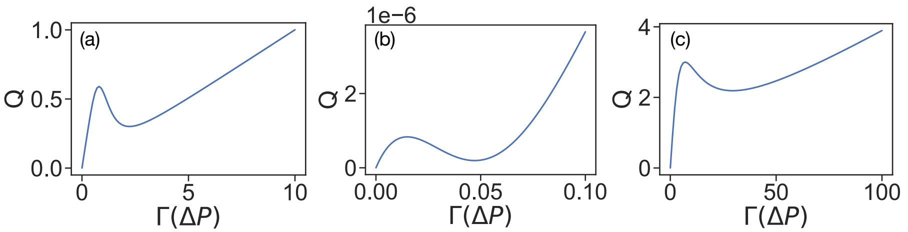

Fig. 1 | Different expressions for $\Gamma_{\mathrm{NL}}(\Delta P)$ used in the main text. Panel (a) shows the expression used in Figure 2 of the main text, following equation (1) in this document. Panels (b) and (c) show the $\Gamma_{\mathrm{NL}}(\Delta P)$ used in figures 3 and 4 of the main text, following equations (2) and (3) here, respectively.

Supplementary Note 1. Mathematical expressions for the nonlinearities

In the main text we solve the equations for the phenomenological mathematical model several times, using different $\Gamma_{\mathrm{NL}}(\Delta P)$. In figure 2a of the main text we plot the functional form of $\Gamma_{\mathrm{NL}}(\Delta P)$, that corresponds to the equation:

$$\Gamma_{\mathrm{NL}} = \Delta P \frac{1 + 0.1(\Delta P)^4}{1 + (\Delta P)^4}. \tag{1}$$

In the case of Figure 3e, we use a function $\Gamma_{\mathrm{NL}}(\Delta P)$ that has been fitted to the behavior of one isolated valve, the expression is

$$\Gamma_{\mathrm{NL}} = \begin{cases} a\Delta P - b(\Delta P)^2 + c(\Delta P)^3 - d(\Delta P)^4 + e(\Delta P)^5 & \text{if } (\Delta P) \geq 0, \\ -\left(-a\Delta P - b(\Delta P)^2 - c(\Delta P)^3 - d(\Delta P)^4 - e(\Delta P)^5\right) & \text{if } (\Delta P) < 0, \end{cases} \tag{2}$$

where $a = 0.0001$, $b = 0.007$, $c = 0.1$, $d = 0.9$ and $e = 2$.
Finally, in Figure 4c we use again the phenomenological model to compare the results with the experimental case. We fitted $\Gamma_{\mathrm{NL}}(\Delta P)$ to the behavior of one experimental valve, the expression used in this case is,

$$\Gamma_{\mathrm{NL}} = a_1 \frac{\Delta P}{d_1} \frac{b_1 + c_1(\frac{\Delta P}{d_1})^2}{1 + (\frac{\Delta P}{d_1})^2}, \tag{3}$$

where $a = 8$, $b = -0.7$, $c = -0.03$, $d = -6.4$. These three expressions for $\Gamma_{\mathrm{NL}}(\Delta P)$ are depicted in figure 1.

Supplementary Note 2. Stability of one valve with negative differential resistance

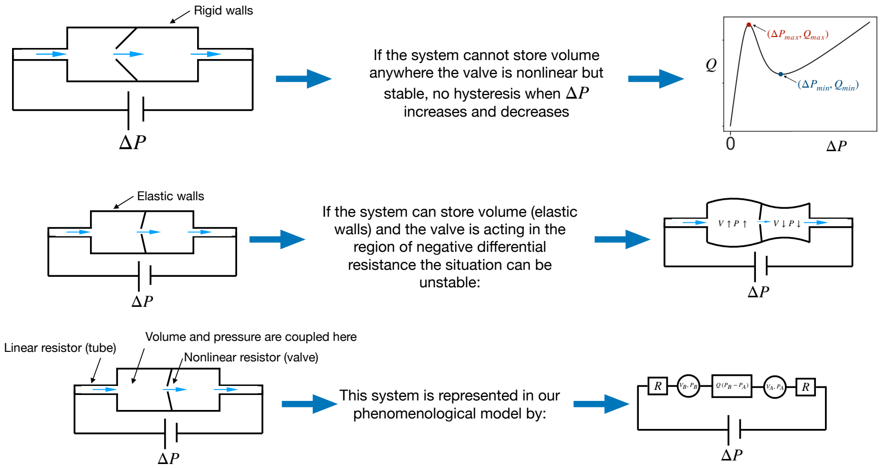

Fig. 2 | Explanation of the stability of one valve with a region of negative differential resistance, when it is connected to an external pump through two linear resistors. If the walls of the system are elastic and can store volume the valve can escape the NDR region and move to the second branch of positive differential resistance. However, this only occurs for a certain range of the resistance of the linear resistors.

We discuss here the stability of a minimalist system where one valve with a region of negative differential resistance is connected to a pump through two linear resistors (rigid tubes). The pump imposes an external pressure difference to the complete system (ΔP). If the system cannot store volume anywhere, the valve will not present any instability. In other words, if we increase and then decrease ΔP the flow through the system does not present any bistability or hysteresis. This is because this situation does not allow any mismatch of the flows within the system.

However, if the walls of the tubes containing the fluid right before and after the valve can store volume, then the NDR region can be unstable. Let us study the condition for which this system can be unstable. The pump imposes an external constraint for the pressure drops inside the system:

$$\Delta P = \Delta P_{NL} + 2\Delta P_L, \tag{4}$$

where ΔP_{NL} and ΔP_L are the pressure drops at the nonlinear resistor (valve) and linear resistors, respectively. Since there are two regions that can store volume due to elastic walls, see Fig. 2, mass conservation controls the volume in these two regions:

$$\begin{cases} \frac{\mathrm{d}V_B}{\mathrm{d}t} &= \frac{\Delta P_L}{R} - Q(\Delta P_{NL}) \\ \frac{\mathrm{d}V_A}{\mathrm{d}t} &= Q(\Delta P_{NL}) - \frac{\Delta P_L}{R} \end{cases} \rightarrow \frac{\mathrm{d}(V_B - V_A)}{\mathrm{d}t} = 2\left(\frac{\Delta P_L}{R} - Q(\Delta P_{NL})\right), \tag{5}$$

where V_B and V_A are the volumes in the regions before and after the valve, see Fig. 2. We also know that volume and pressure are coupled, in our model we consider

elastic walls that couple volume with pressure as:

$$V_i = 1 - \alpha(P_{i+1} - 2P_i + P_{i-1}), \tag{6}$$

in this simple system, this leads to:

$$V_B - V_A = \alpha(-\Delta P + 3P_B - 3P_A) \to \frac{\mathrm{d}(V_B - V_A)}{\mathrm{d}t} \propto \frac{\mathrm{d}(P_B - P_A)}{\mathrm{d}t}, \tag{7}$$

it is clear that other simpler couplings such as $V_i \propto P_i$ will also lead to the same result. Combining now equations (5) and (7):

$$\frac{\mathrm{d}(P_B - P_A)}{\mathrm{d}t} \propto 2\left(\frac{\Delta P_L}{R} - Q(\Delta P_{NL})\right), \tag{8}$$

using now that $\Delta P_{NL} = (P_B - P_A)$ and equation (4), we get,

$$\frac{\mathrm{d}\Delta P_{NL}}{\mathrm{d}t} \propto 2\left(\frac{\Delta P - \Delta P_{NL}}{2R} - Q(\Delta P_{NL})\right). \tag{9}$$

Let's consider that the system is at a stationary solution with a pressure difference at the valve ΔP^*_{NL} that is within the region of negative differential resistance,

$$\frac{\mathrm{d}\Delta P^*_{NL}}{\mathrm{d}t} = 0, \tag{10}$$

then, we are interested in the evolution of a small perturbation,

$$\frac{\mathrm{d}\Delta P^*_{NL} + \epsilon}{\mathrm{d}t} \propto 2\left(\frac{\Delta P - \Delta P^*_{NL} - \epsilon}{2R} - Q(\Delta P^*_{NL} + \epsilon)\right), \tag{11}$$

linearizing the flow through the valve around ΔP^*_{NL} we get,

$$\frac{\mathrm{d}\epsilon}{\mathrm{d}t} \propto -2\left(\frac{1}{2R} + Q'(\Delta P^*_{NL})\right)\epsilon, \tag{12}$$

where $Q'(\Delta P^*_{NL})$ is the derivative of $Q(\Delta P_{NL})$ evaluated at ΔP^*_{NL}. Since ΔP^*_{NL} is in the NDR region, $Q'(\Delta P^*_{NL}) < 0$. The stationary solution will be unstable if,

$$\left(\frac{1}{2R} + Q'(\Delta P^*_{NL})\right) < 0, \tag{13}$$

what leads to,

$$\frac{1}{2R} < -Q'(\Delta P^*_{NL}), \tag{14}$$

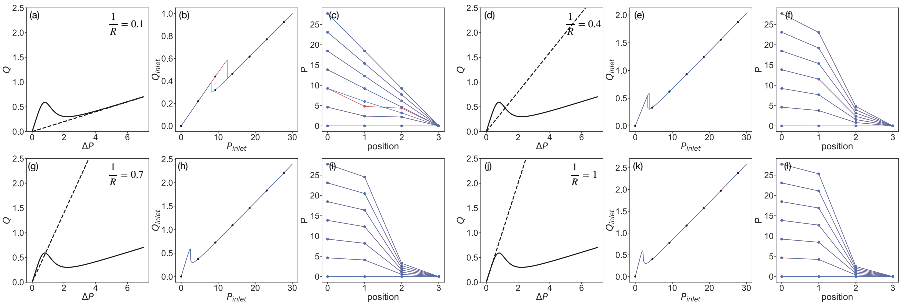

Fig. 3 | Numerical stability analysis for a NDR valve within a minimalist system containing two linear resistors and two regions that can accumulate volume (nodes) connected to an external pump, see Fig. 2 for a squeme of the system. Instabilities lead to the bistable behavior of the system for $\frac{1}{R} < 0.4$, as the theory predicts. For each simulation we show three panels. Panels (a,d,g,j) display flow versus pressure difference for the nonlinear valve (continuous line) and the linear resistor (dashed line). Panels (b,e,h,k) show the flow through the complete system versus the external pressure imposed at the inlet by the pump, red and blue lines indicate increasing and decreasing pressure difference, respectively. Finally, panels (c,f,i,l) present the pressure in the four nodes of the system at different times (marked in panels (b,e,h,k) as black dots.

approximating the derivative in the NDR by a straight line going through the local maximum and minimum (see Fig. 2), we get,

$$\frac{1}{R} < \frac{2\left(Q_{max} - Q_{min}\right)}{\left(\Delta P_{min} - \Delta P_{max}\right)}. \tag{15}$$

To test this result we have carried out some simulations with $\alpha = 0.001$ (volumes will be close to one) and

$$\Gamma_{\rm NL} = \Delta P \frac{1 + 0.1(\Delta P)^4}{1 + (\Delta P)^4}, \tag{16}$$

for this case (15) gives a stability threshold $\frac{1}{R} \approx 0.4$. Indeed when we carry out the simulations we see that linear resistors with a larger resistance than the threshold lead to bistability, whereas a system with linear resistors with smaller resistance do not show any unstable behavior, see Fig. 3. We have also validated this effect in the experimental setup, Fig. 4. In this case a low linear resistor (R1) leads to a very small bistability region where a larger linear resistance (R2) leads to a larger bistability region as the theory predicts.

Supplementary Note 3. Stability of homogeneous stationary profiles

Let us discuss here the stability of a homogeneous solution to the system of equations considered in the main text. Given a 1D network of NDR resistors connected in series,

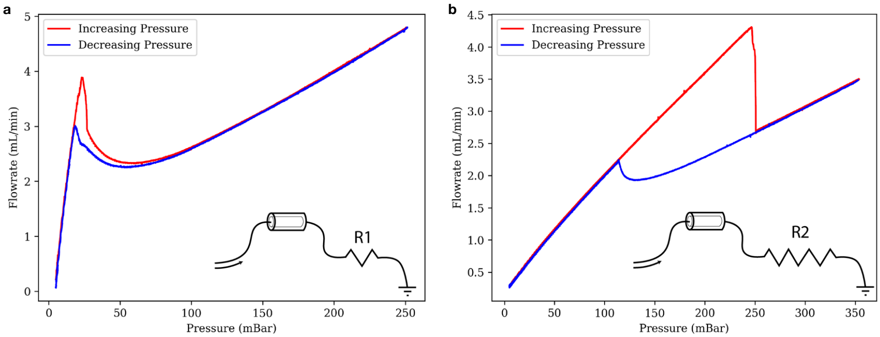

Fig. 4 | Experiments with one NDR valve within a flexible tube connected with a linear resistor. **a** shows the flow through the system when the linear resistor has a low resistance (R1). **b** presents the same case for a linear resistor of larger resistance (R2). As the theory predicts, a larger linear resistance leads to bistability.

a constant pressure drop at every edge, $\Delta P_i = \Delta P^*$ and $V_i = 1$, is a stationary solution of the system. For simplicity lets consider here a generic expression for the current $Q(\Delta P_i)$ from node i to $i + 1$ that only depends on the pressure difference between the two nodes. We also use the coupling between pressure and volume, which in the 1D network takes the form,

$$V_i - 1 = \alpha(\Delta P_i - \Delta P_{i-1}). \tag{17}$$

Consider now a small perturbation around the stationary state,

$$\Delta P_i = \Delta P^* + \epsilon \Delta p_i, \text{ and } V_i = 1 + \epsilon v_i. \tag{18}$$

Substituting these expressions into Eq. (17) we get,

$$v_i = \alpha(\Delta p_i - \Delta p_{i-1}). \tag{19}$$

The conservation of volume in the system is given by

$$\frac{dV_i}{dt} = Q(\Delta P_{i-1}) - Q(\Delta P_i). \tag{20}$$

Linearizing Q around ΔP^* and using (18) and (19) we get

$$\frac{dv_i}{dt} = -\frac{Q'(\Delta P^*)}{\alpha} v_i. \tag{21}$$

Now it is clear that region a negative slope of $Q(\Delta P)$ at ΔP^* will result in an exponential increase of the small perturbations of the accumulated volume (v_i). This is the basic mechanism that renders some of the "trivial" stationary solutions of the

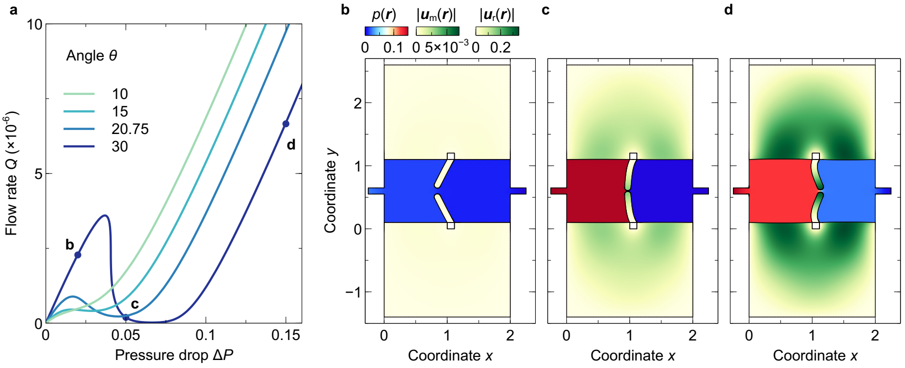

Fig. 5 | Valve geometry controls the steady-state nonlinear response. **a**, Dimensionless steady-state flow rater Q as a function to the applied pressure drop ΔP as a function of the angle at which the elastic rods are clamped with respect to the vertical. **b**-**d**, Dimensionless steady-state pressure field and displacement moduli of the elastic rods and outer medium at different pressure drops, indicated with labels in panel a.

model unstable when the current versus pressure drop presents a region of negative slope. For more details see [1].

Supplementary Note 4. Finite elements simulations

We perform full numerical simulations of the system of equations of the Main Text [Eqs.6-12] using the finite-element method, which involves writing the equations in weak form by means of integral scalar products using test functions for the fluid velocity field, and the displacements fields of the elastic medium and rods. Using Green identities, we obtain an integral bilinear system of equations for the variables and their test functions that is solved using a Newton-Raphson algorithm. To ensure numerical stability, the equations are discretized in space using second-order Lagrange polynomials and triangular elements for the fields, and evolved in time through a 4th-order variable-step backward differentiation formula method. The relative tolerance of the nonlinear method is always set below 10^{-6}. The time-dependent solver was complemented with an automatic remeshing algorithm to capture the accurately solve the displacement of the elastic rods. More details of the numerical techniques can be found in Ref. [2]. As an example, we show in Fig. 5 the flow versus pressure difference relationships for several valves with different geometries.

Supplementary Note 5. Soft valves respond differently to pressure difference depending on the absolute pressure

In the paper, there is a good qualitative agreement between the 1D model and the realistic simulations or the experiments: the model explains all the fundamental mechanisms behind the nested hysteresis loops, the slopes of the branches, etc.

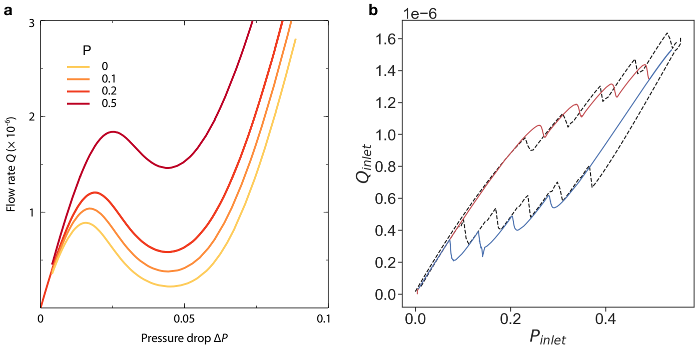

Fig. 6 | a Simulations with a soft valve presenting the flow versus pressure difference as the pressure at the inlet and outlet are shifted an equal amount P. **b** Comparison between realistic simulation (solid line) and phenomenological model (dashed line) using $\Gamma(\Delta P, P)$, for a system with 8 valves.

However, there is a quantitative difference, the upper and bottom branches of the hysteresis loop in the realistic simulations and experiments are tilted upwards, whereas they are horizontal in the 1D model.
In the 1D model we assume that the flow through the soft valves only depends on the pressure difference across the valve, in other words $\Gamma(\Delta P)$ does not depend on P (the absolute pressure at the entrance of the valve). We test here this assumption carrying out simulations with the same soft valve that we used in Fig. 3 of the main text. We use only one valve here and we impose a inlet pressure $\Delta P + P$ and an outlet pressure of P, where $P = 0,\ 0.1,\ 0.2,\ 0.3,\ 0.4,\ 0.5$. The results of these simulations are presented in Fig. 6a, they show how as P increases the flow is shifted upwards, what can explain the tilting of the hysteresis loop. To incorporate this effect into the 1D model one could use a function Γ that not only depends on ΔP but also on the absolute pressure P.
Note that the curves in Fig. 6a are not multiplied by a simple factor that depends on P, for example, the flow at the local maximum increases by a factor of 2 when P goes from 0 to 0.5 whereas the flow at the local minimum increases approximately by a factor of 5. To include this effect in a simple way we multiply $\Gamma_{NL}(\Delta P)$ in equation (2) by a linear factor that depends on ΔP and P, leading to a new $\Gamma(\Delta P, P)$:

$$\Gamma(\Delta P, P) = (1 + (C_1 + C_2 \Delta P)P)\Gamma_{NL}(\Delta P) \tag{22}$$

where C_1 and C_2 are constants that are adjusted so that the flow at the local maximum and minimum of $\Gamma(\Delta P, P)$ are multiplied by a factor 2 and 5, respectively, when P goes from 0 to 0.5, leading to $C_1 = -0.8$ and $C_2 = 187$.
We now use this $\Gamma(\Delta P, P)$ in the phenomenological model and carry out a simulation with the same system as in Fig. 3 of the main text. Fig. 6b shows how with only this simple expression for $\Gamma(\Delta P, P)$ the phenomenological model agrees quantitatively with the realistic simulation.

References

[1] Ruiz-García, M. & Katifori, E. Emergent dynamics in excitable flow systems. *Physical Review E* **103** (6), 062301 (2021) .

[2] Martínez-Calvo, A., Sevilla, A., Peng, G. G. & Stone, H. A. Start-up flow in shallow deformable microchannels. *J. Fluid Mech.* **885** (2020) .
